# Bridging herb and needle: shared hepatoprotective mechanisms of saikosaponins and acupuncture and the rationale for combined therapy

**DOI:** 10.3389/fmolb.2026.1949938

**Published:** 2026-10-14

**Authors:** Yu Xu, Lujun Tang, Min Lai, Yanmei Yang, Lu Zhang, Xiaoting Tian

**Affiliations:** 1 Chongqing College of Traditional Chinese Medicine (a. School of Traditional Chinese Medicine; b. School of Chinese Materia Medica), Chongqing, China; 2 School of Traditional Chinese Medicine, Chongqing Medical and Pharmaceutical College, Chongqing, China

**Keywords:** acupuncture, hepatoprotection, integrative medicine, liver fibrosis, Radix Bupleuri, saikosaponins

## Abstract

Liver disease remains a major global health burden, and treatments that directly reverse established fibrosis remain limited and indication-specific. Radix Bupleuri was selected for this review because it is widely used in traditional East Asian liver-directed formulas and because its principal triterpenoid constituents, saikosaponins, have a substantial but predominantly preclinical hepatology literature. This review critically synthesizes saikosaponin evidence across chemical, immune-mediated, alcohol-related, cholestatic, fibrotic, and metabolic injury, emphasizing congener-specific actions, experimental-model limitations, poor bioavailability, dose-dependent hepatotoxicity, and potential cytochrome-mediated interactions. We separately appraise manual acupuncture, electroacupuncture, moxibustion, and acupoint catgut embedding. Human evidence is strongest for metabolic liver disease and is concentrated in electroacupuncture and embedding studies; evidence for fibrosis and cirrhosis is weaker, while most mechanistic data derive from electroacupuncture or moxibustion in rodents. The two evidence streams show partial functional overlap in fibrogenic cell state and matrix turnover, redox control, innate-inflammatory signaling, and metabolic-autophagic regulation. This overlap is asymmetric: vagus-dependent hepatic Nrf2 activation is supported for electroacupuncture, whereas direct evidence for canonical hepatic Nrf2 activation by saikosaponins remains limited. Gut microbiota, bile-acid metabolism, intestinal-barrier integrity, and gut-brain signaling form an additional systems-level interface, but their causal role in a combined intervention is untested. No direct co-administration study was identified. The proposed combination should therefore be treated as a falsifiable research hypothesis, not as demonstrated synergy or an established therapy.

## 1 Introduction

Liver disease imposes a large and rising global burden. It causes about two million deaths each year, roughly 4% of all deaths worldwide (Devarbhavi et al., 2023). Most chronic liver injury follows a shared course whatever its cause, whether viral hepatitis, alcohol or metabolic dysfunction. This course runs from hepatocyte injury to fibrosis, then cirrhosis and ultimately hepatocellular carcinoma (HCC) (GBD, 2017 Cirrhosis Collaborators, 2020). The parallel rise of non-alcoholic fatty liver disease, now affecting close to a third of the world’s population, has further enlarged the group at risk of fibrotic progression (Amini-Salehi et al., 2024; Younossi et al., 2023). Across these conditions, activation of hepatic stellate cells (HSCs) is widely seen as the central driver of fibrogenesis. In response to injury, quiescent HSCs transdifferentiate into proliferative, contractile myofibroblasts that deposit excess extracellular matrix (ECM) (Tsuchida and Friedman, 2017). Approved antifibrotic therapies remain scarce, and current management is largely aetiology-directed (Bataller and Brenner, 2005). A safe, mechanistically grounded hepatoprotective strategy is therefore still needed.

Radix Bupleuri was chosen as the herbal focus for three linked reasons. First, *Bupleurum chinense* DC. and *Bupleurum scorzonerifolium* Willd. are the principal botanical sources recognised for Chaihu in Chinese medicine, and Chaihu has a long history of use in formulas directed at disorders interpreted as involving constrained liver qi, inflammatory syndromes, and chronic hepatic disease (Yang et al., 2017). Second, saikosaponins are among the best-characterised chemical constituents of Radix Bupleuri, allowing formula-level traditional use to be separated from congener-specific pharmacology (Ashour and Wink, 2011; Yuan et al., 2017). Third, the available literature spans efficacy, molecular mechanism, pharmacokinetics, formulation, and toxicity, making the plant a useful case for a balanced translational review rather than a catalogue of favourable findings. Evidence from Xiao-Chai-Hu-Tang, Chaihu-Shugan-San, Xiaoyao-San, or other multicomponent prescriptions is discussed as contextual traditional-Chinese-medicine evidence and is not attributed to an isolated saikosaponin (Kong et al., 2019; Xie et al., 2022; Qu et al., 2025).

For saikosaponins, a broad preclinical literature documents protection across chemically induced, immune, alcoholic, cholestatic and metabolic models of liver injury. The benefit is traced to suppression of HSC activation, oxidative stress and inflammatory signalling, together with regulation of lipid metabolism and autophagy. This picture is not uniformly favourable. Saikosaponins have a well-recognised dual, dose-dependent nature and can themselves provoke hepatotoxicity under certain conditions (Fan et al., 2025; Zhu et al., 2026), a caveat central to any translational appraisal. For acupuncture, clinical evidence is strongest in metabolic liver disease. A randomised controlled trial, for example, reported that electroacupuncture reduced hepatic steatosis measured by MRI-PDFF in non-alcoholic steatohepatitis (Zhao et al., 2023). Mechanistic work has meanwhile defined specific neuro-immune circuits, notably the somatosensory-driven vagal–adrenal axis engaged by stimulation at ST36 (Liu Q. et al., 2021). Yet rigorous randomised trials of acupuncture for liver fibrosis and cirrhosis specifically remain scarce, and much mechanistic insight comes from electroacupuncture or moxibustion in animal models.

Despite these parallel advances, the two lines of enquiry have developed apart, and whether they share convergent hepatoprotective mechanisms has never been examined systematically. Existing reviews address saikosaponins or acupuncture separately. Evidence syntheses of traditional therapies for liver disease usually treat herbal and physical interventions as distinct categories rather than mechanistically related ones (Liu C. et al., 2025). This separation is partly historical, because the two traditions are studied by different communities using different methods, molecular pharmacology on one side and clinical and neurophysiological research on the other. The cost is a missed question, namely, whether their independently documented benefits reflect action on the same disease machinery. Critically, no direct experimental or clinical study has yet tested saikosaponins and acupuncture in combination, leaving their integrative potential unexplored.

This review compares two distinct evidence streams rather than assuming that a traditional herbal intervention and a physical intervention are interchangeable. Section 2 critically evaluates saikosaponin efficacy, congener specificity, experimental models, pharmacokinetics, delivery, and toxicity. Section 3 distinguishes manual acupuncture, electroacupuncture, moxibustion, and acupoint catgut embedding and separates clinical metabolic outcomes from predominantly preclinical mechanistic claims. Section 4 then asks whether partially overlapping downstream effects justify direct interaction experiments. Throughout, functional overlap is treated as a source of testable questions, not as evidence of an identical molecular mechanism, additivity, synergy, or clinical benefit.

Literature was identified by searching PubMed, Web of Science and the China National Knowledge Infrastructure from database inception to 2026. The search combined the terms *saikosaponin*, *Radix Bupleuri*/*Bupleurum*, *Chaihu*, *acupuncture*, *electroacupuncture*, *moxibustion* and *acupoint* with *liver*, *hepatoprotect,* hepatic fibrosis*,* NAFLD*/*NASH*,* cirrhosis *and* hepatocellular carcinoma*. Original mechanistic studies, randomised trials, meta-analyses and authoritative reviews were prioritised. Primary research was cited in preference to reviews for specific mechanistic claims, and retracted records were excluded.

## 2 Saikosaponin-based interventions in hepatoprotection

Saikosaponins are oleanane-type triterpenoid glycosides and a major bioactive fraction of Radix Bupleuri. The best-studied congeners are saikosaponin a (SSa) and saikosaponin d (SSd), followed by SSb1 and SSb2. Reported hepatoprotective activity spans several injury contexts, but the evidence is predominantly preclinical and cannot be interpreted as a uniform class effect. Congener, dose, formulation, disease model, cell system, exposure duration, and the direction of autophagic regulation all influence the observed outcome. The following synthesis therefore evaluates efficacy together with experimental limitations, pharmacokinetics, and toxicity.

### 2.1 Hepatoprotective activities across liver injury models

Saikosaponin studies cover chemical, immune-mediated, metabolic, alcohol-related, cholestatic, and fibrotic models, but the breadth of models should not be mistaken for equivalent evidence. In carbon-tetrachloride (CCl4) injury, SSd and SSa reduced lipid peroxidation, transaminases, oxidative stress, and NLRP3-related responses (Abe et al., 1982; Wu et al., 2008; Chen et al., 2020). SSa also attenuated LPS/D-galactosamine injury through LXRalpha-associated signaling (Zhu et al., 2019). In thioacetamide injury and high-fat-diet fatty liver, saikosaponin treatment improved biochemical or histological measures, while lipidomic and transcriptomic studies linked SSa and SSd to lower Fasn/Acaca and higher Acox1/Cpt1a expression (Chang et al., 2021; Li H. et al., 2021). SSd affected PPARalpha/INSIG/SREBP-1c signaling in metabolic fatty liver, saikogenin A affected SIRT1-mTOR-PPARalpha signaling in alcohol-related steatosis, and SSd altered gut microbiota-bile-acid metabolism in NAFLD models (Gu et al., 2022; Jiang et al., 2024; Li et al., 2024). These results identify plausible pathways, but model-specific outcomes, short treatment periods, heterogeneous doses, and limited replication constrain cross-model inference. CCl4, thioacetamide, and LPS/D-galactosamine reproduce selected injury processes rather than the full natural history of human chronic liver disease; diet models better represent metabolic stress but vary in inflammation and fibrosis. Cell-culture findings require additional caution. For example, HL-7702/L-02 has been reported to be cross-contaminated or HeLa-related, so the Lin et al. (2018) result should not be treated as definitive evidence from normal human hepatocytes. Formula-level clinical findings are also not compound-level evidence: in the Sho-saiko-to study, the overall hepatocellular-carcinoma comparison did not reach conventional significance (P = 0.071), and the significant result was confined to an HBsAg-negative subgroup (Oka et al., 1995). Overall, as summarized in Table 1, the literature supports congener- and context-dependent biological activity, not a single established hepatoprotective mechanism or clinical effect.

**TABLE 1:** Hepatoprotective activities of saikosaponins across liver injury models: congeners, mechanistic nodes and key findings.

Saikosaponin	Model	Mechanistic targets	Results
SSd	CCl_4_(acute/chronic)	Redox; HSC	↓ lipid peroxidation; attenuated cirrhotic change
SSa	CCl_4_	Redox	↑ SOD/GSH; ↓ transaminases
SSd	CCl_4_hepatitis	Redox; inflammation (NLRP3)	↓ oxidative stress and NLRP3 activation
SSa	LPS/D-GalN	Inflammation (LXRα)	↓ acute immune-mediated injury
SSd	TAA + HFD-NAFLD	Metabolism	↓ transaminases and histological score in both models
SSa/SSd	HFD-NAFLD	Metabolism (lipidome)	↓ Fasn/Acaca; ↑ Acox1/Cpt1a
SSd	MAFLD	Metabolism (PPARα/SREBP1c)	↓ hepatic steatosis
Saikogenin A	Alcoholic steatosis	Metabolism (SIRT1–mTOR)	↓ steatosis
SSd	Cholestasis	Inflammation (TLR4/NLRP3–ER stress)	↓ injury
SSd	Activated HSCs (*in vitro*)	HSC apoptosis (caspase/mitochondrial)	Induced apoptosis of activated HSCs
Sho-saiko-to (formula)	Cirrhosis patients	Clinical outcome	Overall HCC comparison P = 0.071; significant only in HBsAg-negative subgroup
SSd	DMN fibrosis	HSC/fibrogenic	↓ collagen I; ↓ TGF-β1
SSd	Hepatic fibrosis	HSC (ERβ/NLRP3)	↓ HSC activation
SSb1	Hepatic fibrosis	HSC (STAT3/Gli1); lower toxicity	↓ fibrosis
SSd (adverse interaction)	Otherwise subtoxic APAP regimen	Autophagy (GABARAP)	Potentiated APAP hepatotoxicity

Abbreviations: SSa/SSb1/SSd, saikosaponin a/b1/d; CCl_4_, carbon tetrachloride; LPS/D-GalN, lipopolysaccharide/D-galactosamine; TAA, thioacetamide; HFD, high-fat diet; NAFLD/MAFLD, non-alcoholic/metabolic-associated fatty liver disease; DMN, dimethylnitrosamine; APAP, acetaminophen; HSC, hepatic stellate cell; HCC, hepatocellular carcinoma; HBsAg, hepatitis B surface antigen.

### 2.2 Structure–activity relationships and congener-specific pharmacology

The pharmacology summarised above is not a class-wide constant. It varies with the fine structure of each congener, and this variation shapes both efficacy and toxicity. Saikosaponins share an oleanane-type triterpenoid skeleton, but they differ in the configuration of the ring system and in their sugar chains, and these differences define the major subtypes (Ashour and Wink, 2011; Yuan et al., 2017). SSa and SSd carry a 13,28-epoxy ether bridge, whereas SSb1 and SSb2 are ring-opened homoannular diene isomers formed from SSa and SSd during processing (Jia et al., 2022). Such structural detail is not merely descriptive. It maps onto distinct molecular targets and distinct safety profiles.

The clearest example is the divergence between SSd and the newer congener SSb1. SSd acts largely through the ERβ axis and the ROS/NLRP3 inflammasome to deactivate stellate cells (Que et al., 2018; Zhang et al., 2022), and it also engages STAT3 and NF-κB in hepatocytes and macrophages (Liu et al., 2014). SSb1, by contrast, attenuates fibrosis by disrupting the STAT3/Gli1 interaction and promoting Gli1 degradation, and it does so with lower hepatocyte toxicity than SSd (Shao et al., 2025). SSa and SSd, though closely related, are not interchangeable either. Integrative profiling shows overlapping but non-identical effects on hepatic lipid genes (Li X. et al., 2021), SSa favours LXRα-dependent and TLR4-directed anti-inflammatory routes (Fu et al., 2015; Zhu et al., 2019), and SSb2 acts mainly on the IKK/IκBα/NF-κB cascade (Shin et al., 2019). Even within the CCl4 model, individual congeners reproduce protection through partly separate antioxidant and inflammasome mechanisms (Lin et al., 2018).

Deglycosylation adds a further layer. Gut microbial and hepatic metabolism converts the parent saponins into secondary glycosides and aglycones, and one such metabolite, saikogenin A, retains activity by relieving alcoholic steatosis through SIRT1 signalling (Jiang et al., 2024). Because these metabolites differ from the parent compounds in potency, target profile and cytochrome interaction, the active species *in vivo* may not be the administered one. Taken together, these data argue that saikosaponin hepatoprotection is congener-specific rather than uniform (Jia et al., 2022; Zhu et al., 2026). These target differences are matched by differences in potency and selectivity, so a congener that is more active against one node is not necessarily the safest or the most selective overall. The practical consequence is direct. The choice of which molecule to advance, and at what dose, cannot be generalised across the class, and it should be guided by the target and the toxicity profile of the specific congener.

### 2.3 Oxidative stress, inflammation and fibrogenic responses

The antifibrotic action of saikosaponins rests on the deactivation of HSCs and on interrupting the oxidative and inflammatory signalling that sustains their activation. In dimethylnitrosamine-induced fibrosis, SSd reduced collagen I deposition and TGF-β1 expression while limiting hepatocyte injury (Fan et al., 2007), and SSa suppressed chemically induced inflammation and matrix accumulation in parallel (Wu et al., 2010). One coherent theme is the oestrogen-receptor-β (ERβ) axis. SSd suppressed oxidative-stress-driven HSC activation in an ERβ-dependent manner (Que et al., 2018) and protected against fibrosis by negatively regulating the NLRP3 inflammasome downstream of ERβ (Lin et al., 2021). Studies in ERβ-knockout mice placed this on firmer causal footing: SSd curbs the ROS/NLRP3 inflammasome specifically through ERβ, and in TGF-β-stimulated LX-2 cells it lowered Col1α1, α-SMA and NLRP3 (Zhang et al., 2022). The anti-inflammatory reach is broader than the inflammasome alone. SSa prevents TLR4 from moving into membrane lipid rafts, which blunts oxidative and inflammatory responses (Fu et al., 2015). It does so by depleting membrane cholesterol through LXRα, and this in turn restrains NF-κB and IRF3 (Wei et al., 2016). Other congeners act on the same cascade. SSb2 inactivates the IKK/IκBα/NF-κB cascade in stimulated macrophages (Shin et al., 2019), while SSd suppresses NF-κB and STAT3 signalling to mitigate acetaminophen hepatotoxicity (Liu et al., 2014) and downregulates COX2 via a p-STAT3/C/EBPβ pathway in liver cancer cells (Ren et al., 2019). SSb1 extends this map to further oleanane-type congeners, attenuating fibrosis by disrupting the STAT3/Gli1 interaction and promoting Gli1 degradation (Shao et al., 2025).

These actions are best understood against the canonical biology of HSC activation. Quiescent cells transdifferentiate into myofibroblasts under the master profibrogenic control of TGF-β/Smad signalling, and the balance between different Smad phosphoisoforms shapes whether the response is fibrogenic or growth-suppressive (Friedman, 2004; Hernandez-Gea and Friedman, 2011; Yoshida et al., 2014). Saikosaponins intersect this programme at several points. They dampen the oxidative and inflammatory inputs that drive Smad-dependent transcription, lower TGF-β1 itself, and, for individual congeners, engage discrete effectors such as ERβ, STAT3/Gli1 or the NLRP3 inflammasome. Structurally similar saponins act through partly distinct nodes and differ markedly in hepatocyte toxicity. This congener-level divergence, set out in Section 2.2, again shows that the class cannot be treated as pharmacologically uniform (Jia et al., 2022).

### 2.4 Metabolic homeostasis, mitochondrial regulation and autophagy

A second mechanistic axis works through the regulation of metabolism, mitochondrial integrity and autophagic flux, and here saikosaponin biology is most nuanced. Autophagy is modulated in a context-dependent direction. In fibrosis, SSd upregulated the membrane oestrogen receptor GPER1 to restrain HSC autophagy, reducing BECN1 and raising p62, with a corresponding fall in Col1 and α-SMA (Chen et al., 2021). In other settings, however, it promoted autophagosome formation to drive HSC apoptosis (Jiang et al., 2021). This divergence reflects the opposing roles autophagy plays in surviving hepatocytes versus activated HSCs, and it warns against treating “autophagy modulation” as a single directional effect. Mitochondrial regulation is a further recurring node. SSd alleviated hepatic ischaemia–reperfusion injury by inhibiting mitophagy-associated ferroptosis through a STAT3/PINK1 pathway (Liu et al., 2026). The metabolic reprogramming described above, namely, PPARα and SREBP1c tuning (Gu et al., 2022), lipogenic-to-oxidative gene shifts (Li H. et al., 2021), and SIRT1–mTOR–PPARα signalling (Jiang et al., 2024), places mitochondrial fatty-acid oxidation at the centre of the metabolic benefit. Evidence from extrahepatic stellate cells, in which SSd inhibited autophagy via the PI3K/Akt/mTOR pathway, offers a parallel for mTOR involvement, but it derives from a pancreatic model and should be extrapolated to the liver only with caution (Cui et al., 2019). Across these studies the metabolic and the autophagic actions are tightly linked, because AMPK and mTOR sit at the junction of energy sensing and autophagic flux, and this coupling is one reason the direction of the autophagy effect depends so strongly on the cellular context.

### 2.5 Dual effects and safety concerns

Saikosaponin hepatoprotection is bounded by a real capacity for harm, and this duality is the decisive consideration for translation. A comprehensive appraisal of the pharmacological and toxicological literature frames saikosaponins as agents whose benefit is dose-dependent and can reverse into toxicity (Zhu et al., 2026). Mechanistic studies bear this out. At sub-toxic doses, SSd aggravated acetaminophen-induced injury by binding GABARAP and obstructing SNARE-dependent autophagosome maturation (Fan et al., 2025). Saikosaponins also worsened cholangiocyte mitochondrial damage and senescence in cholestasis by inhibiting protective autophagy (Fan et al., 2026), the same autophagic machinery that mediates benefit in other contexts. Drug-interaction liability adds to the concern. Saikosaponins and their deglycosylated metabolites modulate CYP3A4 through the pregnane X receptor (Liu S. et al., 2021), and SSd alters CYP1A2, CYP2D6 and CYP3A4 expression and activity in HepaRG cells, with molecular docking indicating direct binding (Li et al., 2020; Li H. et al., 2021). Because CYP3A4 metabolises a large share of clinical drugs, this creates a realistic interaction risk when saikosaponins are given alongside co-medications. The same enzymes also handle hepatotoxins such as acetaminophen, whose injury SSd can worsen at sub-toxic doses (Fan et al., 2025). The direction of the effect, induction or inhibition, appears to depend on the congener, the dose and the exposure time, so it cannot be assumed to be uniform across the class. These liabilities, together with the poor aqueous solubility and low oral bioavailability of the parent compounds (Sun et al., 2025), sharpen the central translational problem. Hepatoprotection must be secured within a defined therapeutic window while autophagy-directed toxicity and cytochrome-mediated interactions are avoided. Direct high-quality evidence that saikosaponins activate hepatic Nrf2/HO-1 signalling also remains limited. As the next section describes, delivery engineering rather than the parent molecule currently offers the most credible route to a favourable benefit–risk balance.

### 2.6 Pharmacokinetics, bioavailability and delivery engineering

Whether the mechanisms above can be exploited clinically depends on getting enough active compound to the liver safely. Here the parent saponins are poorly served by their own physical chemistry. They have limited aqueous solubility and low oral bioavailability, and systemic exposure after oral dosing is modest (Sun et al., 2025). Native saponins also carry surfactant-like membrane activity that can cause haemolysis, which constrains parenteral use (Zhang et al., 2015). These properties, rather than any lack of intrinsic potency, are a main barrier to translation.

Delivery engineering has become the most active response to this problem. Liposomal co-encapsulation of SSa and SSd reduced haemolysis and improved pharmacokinetic exposure relative to the free compounds (Zhang et al., 2015). In fibrotic mice, a liposomal saikosaponin formulation produced stronger antifibrotic repair with lower toxicity than the unformulated drug, which is the combination a narrow therapeutic window demands (Shiu et al., 2020). More recent designs go further and use SSd itself as a structural membrane component of multifunctional nanocarriers directed at hepatocellular carcinoma, turning the molecule from cargo into part of the vehicle (Yu et al., 2025). Across these studies the recurring gain is the same, namely, a wider gap between the effective dose and the toxic dose.

Two limits temper this promise. First, almost all of the pharmacokinetic and delivery data come from rodents, and human exposure, dosing and tissue distribution remain uncharacterised. Second, formulation changes the disposition and the metabolism of the compound, so a delivery system optimised for exposure must still be checked against the autophagy-directed toxicity and cytochrome interactions described above. Beyond liposomes, other strategies pursue the same goal, including structural modification of the saponin and co-formulation to improve solubility, though these approaches are earlier in development. The decisive missing step is human data, because no formulation has yet been characterised for pharmacokinetics, tissue distribution or safety in patients. For a class whose main liability is a narrow and dose-dependent safety margin, delivery engineering still offers the most credible near-term route to a favourable benefit–risk balance. It also sets a concrete requirement for any future combination study, which is a defined and reproducible saikosaponin formulation rather than a crude extract.

## 3 Acupuncture-based strategies in hepatoprotection

Acupuncture-related interventions are not a single modality. Manual needling, electroacupuncture, moxibustion, and acupoint catgut embedding differ in stimulus, dose, duration, operator dependence, and plausible physiology. Human evidence is concentrated in metabolic liver disease and in electroacupuncture or embedding protocols; moxibustion and most mechanistic pathways are supported mainly by animal studies, while manual acupuncture is underrepresented in controlled liver trials. Accordingly, mechanistic conclusions below name the tested modality and are not generalised automatically to acupuncture as a whole.

### 3.1 Rationale for acupuncture in liver injury and repair

In traditional theory, acupuncture is directed at the liver by regulating qi and blood and resolving stasis, a framework proposed to underlie its antifibrotic potential (Zhou et al., 2012). Contemporary work has supplied a physiological basis for the acupoints most often chosen in liver disease. Needling the classical liver-directed combination of Zusanli (ST36) and Taichong (LR3) lowered serum aminotransferases in rats with CCl4-induced acute liver damage, giving direct experimental support for this pairing (Liu et al., 2001). Moxibustion at the liver back-shu point Ganshu (BL18) reduced serum α-fetoprotein and hepatic Livin in a model of pre-neoplastic liver injury (Jia et al., 2014). The strongest justification for point selection comes from work showing that the somatosensory neurons driving anti-inflammatory circuits are anatomically specific: stimulation at ST36, but not at abdominal points, engages a vagal–adrenal axis through PROKR2-marked sensory neurons (Liu Q. et al., 2021). This reframes acupoint choice as a question of which sensory pathways a site recruits, rather than classical location alone, and it grounds the recurrent use of ST36, LR3 and BL18 in liver protocols. This anatomical view also predicts where point selection should fail. If the anti-inflammatory effect depends on recruiting specific sensory neurons, then points that do not engage those neurons should not reproduce the systemic response, and the intensity of stimulation should matter as much as its location, because the vagal-adrenal circuit is driven only above a threshold of afferent activation (Liu S. et al., 2021). These predictions give acupoint selection a testable physiological basis rather than a purely traditional one, and they bear directly on how the acupuncture arm of a combination study should be designed.

### 3.2 Acupuncture-related interventions and current evidence

Clinical evidence for acupuncture in liver disease is unevenly distributed. It is most convincing in NAFLD and its steatohepatitic form, and progressively weaker in fibrosis and cirrhosis. The most rigorous signal is a randomised controlled trial in which electroacupuncture reduced hepatic fat quantified by MRI-PDFF in non-alcoholic steatohepatitis, outperforming sham stimulation (Zhao et al., 2023). A further randomised trial found that electroacupuncture added to lifestyle modification improved hepatic steatosis and glucolipid metabolism beyond lifestyle change alone in obese NAFLD (Dong et al., 2020). Pooled analyses reinforce this direction while exposing its limits. A systematic review of thirty randomised trials reported that acupuncture raised the overall response rate in NAFLD and that acupoint catgut embedding gave the most stable effect (Liu S. et al., 2025). Separate meta-analyses of acupoint embedding, and of acupoint therapies more broadly, found reductions in transaminases and improvements in lipid profiles (Bi et al., 2021; Dai et al., 2020). The evidence weakens sharply for advanced disease. A meta-analysis of acupuncture as an adjunct in liver cirrhosis pooled fifteen trials but judged the evidence quality low (Qi et al., 2020), and a Cochrane review of acupuncture for chronic hepatitis B found the effect on virological outcomes uncertain and the certainty of evidence very low. Underpinning the clinical signal is a broader base of experimental work across modalities and acupoints. Manual acupuncture at ST36, CV4 and KI1 attenuated steatohepatitis in methionine–choline-deficient mice (Meng et al., 2019), warm acupuncture and moxibustion improved hepatic lipid handling in dietary models (Lee et al., 2024), and electroacupuncture modulated hepatic energy metabolism and the gut microbiota in obese diabetic rats (Ding et al., 2023), as summarised in recent mechanistic reviews (Wang and Zhang, 2026) and collated in Table 2. The modalities are not equivalent, and the evidence is distributed unevenly among them. The strongest human signals come from electroacupuncture and from acupoint catgut embedding, the two forms most used in the NAFLD trials (Bi et al., 2021; Dai et al., 2020; Liu C. et al., 2025), whereas moxibustion and warm acupuncture are supported mainly by mechanistic work in animals (Lee et al., 2024; Xu et al., 2023). Manual needling, the form closest to classical practice, is the least represented in controlled liver trials. This imbalance means that a conclusion drawn for acupuncture as a whole may not transfer to every modality, and it argues for specifying the modality precisely rather than pooling forms as a single intervention. The recurring caveats, namely, small and largely Chinese-language trials, heterogeneous protocols, and scarce sham-controlled fibrosis endpoints, mean that the clinical case rests more securely on metabolic surrogates than on histological reversal of fibrosis.

**TABLE 2:** Acupuncture-based hepatoprotection: modalities, mechanisms and hierarchy of clinical and preclinical evidence.

Modality	Model	Mechanistic targets	Key finding
Electroacupuncture	Pilot RCT, n = 60; NASH	Metabolism	Reduced MRI-PDFF; no significant MRE-stiffness difference
Electroacupuncture + lifestyle	Obese NAFLD	Metabolism	↓ steatosis; improved glucolipid metabolism
Mixed acupuncture modalities	NAFLD	-	↑ overall response rate (30 RCTs)
Acupoint embedding/therapy	NAFLD	Metabolism	↓ transaminases; improved lipids
Acupuncture (adjunct)	Liver cirrhosis	-	Possible adjunctive benefit; low-certainty evidence
Acupuncture	Chronic hepatitis B	-	Virological effect uncertain; very-low-certainty evidence
ST36 + LR3	CCl_4_ acute injury	-	↓ AST/ALT
Electroacupuncture (ST36)	Hepatic I/R	Redox (vagus–Nrf2)	Protection abolished by vagotomy
Electroacupuncture	BDL injury	Inflammation (cholinergic anti-inflammatory)	↓ hepatic damage
Acupuncture	CCl_4_ fibrosis	HSC	↓ α-SMA/collagen I; ↑ MMP-9; ↓ PDGF
Electroacupuncture	T2DM/MAFLD	Metabolism–autophagy (AMPK)	↓ steatosis and fibrogenic signalling
Moxibustion (ST36)	Hyperlipidaemia	Autophagy (AMPK/mTOR)	↑ hepatic autophagy

Abbreviations: NASH, non-alcoholic steatohepatitis; MRI-PDFF, magnetic resonance imaging proton-density fat fraction; RCT, randomised controlled trial; ST36, Zusanli; LR3, taichong; I/R, ischaemia–reperfusion; BDL, bile-duct ligation; T2DM, type 2 diabetes mellitus; α-SMA, α-smooth muscle actin; MMP-9, matrix metalloproteinase-9; PDGF, platelet-derived growth factor.

### 3.3 Mechanisms by which acupuncture promotes hepatoprotection

Mechanistic evidence is strongest for selected electroacupuncture paradigms and should not be transferred uncritically to manual needling, moxibustion, or catgut embedding. ST36 electroacupuncture can recruit anatomically defined somatosensory-autonomic circuits, and vagotomy experiments support a vagus-dependent Nrf2 response in hepatic ischemia-reperfusion injury (Liu Q. et al., 2021; Jiang et al., 2023). In metabolic liver models, electroacupuncture has also been associated with NRF2/ARE, AMPK-dependent glycolipid metabolism, autophagy, and TGF-beta1/Smad changes, whereas ST36 moxibustion has been linked to AMPK/mTOR-regulated hepatic autophagy (Deng et al., 2026; Duan et al., 2024; Xu et al., 2023). Evidence for cholinergic macrophage signaling is less liver-specific than is sometimes implied: alpha7nAChR-JAK2/STAT3 signaling was demonstrated in intestinal macrophages in postoperative ileus, and related visceral models establish biological plausibility rather than direct proof in hepatic macrophages (Yang et al., 2021; Hu et al., 2013; Zhang et al., 2021). Likewise, reductions in whole-liver alpha-SMA, collagen I, or PDGF-associated signaling are consistent with lower fibrogenic activity but do not by themselves prove direct HSC deactivation (Kong et al., 2012; Zhang et al., 2012).

The gut-liver axis provides a systems-level connection between the two evidence streams. SSd has altered gut-microbiota composition and bile-acid metabolism in NAFLD models, while electroacupuncture has affected gut microbiota, intestinal-barrier integrity, and gut-brain metabolic signaling (Ding et al., 2023; Li et al., 2024; Lei et al., 2022; Liu S. et al., 2025). These observations suggest a possible sequence from intervention to microbial or barrier change, altered bile-acid or inflammatory signaling, and hepatic metabolic or immune effects. However, the relevant studies use different diseases, modalities, and endpoints, and few establish mediation with microbiota transfer, depletion, bile-acid-receptor blockade, or other causal tests. The gut-liver axis is therefore incorporated here as a cross-cutting interface rather than declared a fifth shared molecular node. A future combination study should measure microbiome composition, bile-acid profiles, intestinal permeability, and hepatic outcomes together to determine whether this interface contributes to interaction.

The translational standing of acupuncture-related hepatoprotection is therefore uneven. Clinical evidence is comparatively stronger for changes in steatosis and metabolic measures than for fibrosis reversal, cirrhosis outcomes, or liver-specific hard endpoints. Mechanistic evidence is more extensive in rodents and is dominated by electroacupuncture or moxibustion; direct cell-resolved hepatic evidence remains limited. These differences should be preserved when interpreting the literature, because efficacy or mechanism demonstrated for one modality, acupoint set, intensity, or disease model cannot be assumed for another.

### 3.4 Standardisation of acupuncture protocols and methodological considerations

The evidence reviewed above repeatedly meets the same obstacle, which is that acupuncture is hard to standardise. This is not a minor reporting issue. It shapes what the trials can show and whether their results can be reproduced or pooled. An acupuncture intervention is defined by many parameters at once: the points chosen, the modality (manual needling, electroacupuncture, moxibustion or catgut embedding), the depth and manipulation of the needle, the frequency and current of electrical stimulation, the duration of each session, and the number and spacing of sessions. Each of these can alter the physiological response, yet they are described inconsistently across studies.

The consequences are visible in the pooled evidence. Meta-analyses in NAFLD report benefit but rest on small, heterogeneous and largely single-country trials, and they differ in which modality gives the most stable effect (Bi et al., 2021; Dai et al., 2020; Liu C. et al., 2025). For advanced disease the problem is sharper, and reviews in cirrhosis and chronic hepatitis B judged the certainty of evidence low to very low, in part because protocols and controls varied so widely (Qi et al., 2020). Sham control is a particular difficulty, because a credible inert needle is hard to design and any needling may produce non-specific effects. Reporting standards exist to address exactly this. The revised STRICTA guideline, an extension of the CONSORT statement, specifies the acupuncture details that trials should report, including the needling, the treatment regimen and the rationale for point selection (MacPherson et al., 2010). Consistent use of such standards would let studies be compared and replicated rather than read in isolation.

For the present review the point is specific. A combination of saikosaponins and acupuncture can only be tested if the acupuncture arm is fixed as precisely as the drug arm. That means a defined set of points, a defined modality, and defined stimulation and session parameters, all reported in full. Without this, an apparent lack of effect could reflect an under-specified protocol rather than a true absence of benefit, and a positive result could not be reproduced. Protocol standardisation is therefore a precondition for the integrative studies proposed below, not a secondary refinement. It is also the single change most likely to make the existing acupuncture literature comparable across studies and, in turn, fit to support a combination trial.

## 4 Mechanistic overlap and combined therapeutic potential of saikosaponins and acupuncture

Saikosaponins and acupuncture-related interventions enter the biological system through fundamentally different routes. The evidence does not demonstrate a common four-node molecular network. Instead, heterogeneous studies report partially overlapping downstream effects in four functional domains: fibrogenic cell state and matrix turnover, oxidative-stress control, innate-inflammatory signaling, and metabolic-autophagic regulation. A fifth cross-cutting interface, the gut-liver axis, may modify these domains through microbiota, bile acids, barrier integrity, and neuroimmune signaling. The strength and directness of evidence differ by congener, acupuncture modality, disease model, and endpoint. Section 4 therefore maps where interaction experiments are justified and where the evidence remains insufficient.

### 4.1 Common biological targets in hepatoprotection

The first domain is fibrogenic cell state and matrix turnover. Defined saikosaponins affect ERbeta-, STAT3/Gli1-, TGF-beta1-, and autophagy-related processes in HSC or fibrosis models, whereas acupuncture-related studies report lower whole-liver alpha-SMA and collagen I, altered MMP-9, and reduced PDGF-associated signaling (Fan et al., 2007; Que et al., 2018; Kong et al., 2012; Zhang et al., 2012; Shao et al., 2025). These endpoints overlap functionally but do not establish the same upstream target or a direct HSC effect for every modality. The second domain is redox control. Saikosaponin evidence is strongest for ERbeta-linked ROS/NLRP3 regulation; direct evidence for canonical hepatic Nrf2/HO-1 activation remains limited. In contrast, vagus-dependent hepatic Nrf2 activation has been tested causally for electroacupuncture in ischemia-reperfusion injury, and NRF2/ARE activation has been reported in a metabolic liver model (Zhang et al., 2022; Jiang et al., 2023; Deng et al., 2026). This is an asymmetric convergence in outcome, not equivalent evidence for a shared Nrf2 node. The third domain is innate-inflammatory signaling. Saikosaponins can affect NF-kappaB, NLRP3, TLR4, LXRalpha, or STAT3 in specific cellular and liver models, whereas electroacupuncture-related cholinergic pathways produce overlapping anti-inflammatory outcomes; complete causal confirmation in hepatic macrophages is still lacking. The fourth domain is metabolic-autophagic regulation. Saikosaponins influence PPARalpha/SREBP-1c-related lipid handling and regulate autophagy in a cell- and injury-dependent direction, while electroacupuncture and moxibustion engage AMPK-associated metabolic or autophagic pathways. AMPK is therefore not established as a common saikosaponin target.

The gut-liver axis cuts across these four domains rather than forming another equivalent molecular node. Microbiota and bile-acid changes may influence hepatic lipid metabolism, redox balance, and innate immunity; intestinal-barrier changes may alter portal inflammatory exposure; and gut-brain signaling may modify systemic metabolism or autonomic output. Evidence on each side remains preclinical and heterogeneous, so a shared causal pathway cannot yet be claimed. The framework in Figure 1 therefore distinguishes direct intracellular, somatosensory-autonomic, and gut-liver routes and marks functional overlap without implying molecular identity.

**FIGURE 1:**
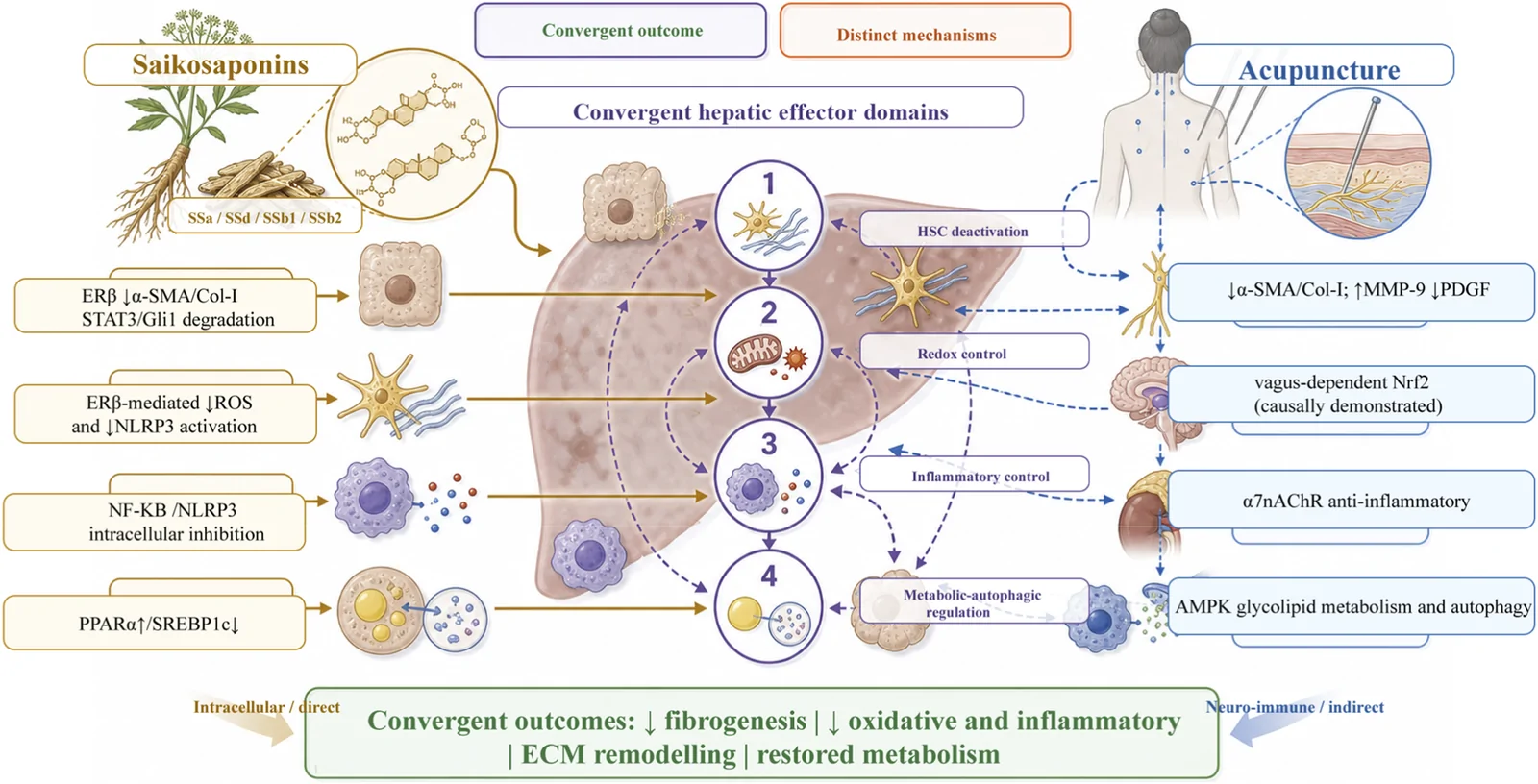
Candidate functional overlap between saikosaponin- and acupuncture-related hepatoprotective evidence. Saikosaponins act mainly through direct cellular pathways, whereas acupuncture-related effects depend on the tested modality and may recruit somatosensory, autonomic, neuroimmune, and gut-liver routes. The evidence streams partially overlap in fibrogenic cell state and matrix turnover, oxidative-stress control, innate-inflammatory signaling, and metabolic-autophagic regulation. The redox evidence is asymmetric: vagus-dependent hepatic Nrf2 activation is supported for electroacupuncture, whereas direct canonical hepatic Nrf2 activation by saikosaponins remains limited. AMPK is not established as a common saikosaponin mechanism. Microbiota, bile acids, intestinal barrier, and gut-brain signaling are presented as a cross-cutting interface requiring causal testing. Functional overlap does not establish molecular identity, additivity, synergy, or clinical efficacy.

### 4.2 Potential benefits and limitations of combined application

The principal value of combining the two approaches is experimental, not therapeutic: distinct upstream routes make it possible to test whether the joint response is additive, sub-additive, antagonistic, or neutral. A four-arm factorial design is required to estimate interaction; pathway overlap alone cannot predict its direction. Claims that acupuncture could lower the necessary saikosaponin dose, widen a therapeutic window, alter hepatic perfusion, or improve target engagement remain untested. Because saikosaponins show dose-dependent hepatotoxicity, context-dependent autophagy effects, poor bioavailability, and potential PXR/CYP liabilities, co-exposure studies must measure plasma and hepatic exposure, liver injury, autophagic flux, and interactions with relevant cytochrome substrates (Liu S. et al., 2021; Fan et al., 2025; Sun et al., 2025; Zhu et al., 2026). Acupuncture-related adverse events are usually mild when treatment is delivered appropriately, but modality-specific and combined safety must still be measured. Redundancy and antagonism are plausible, particularly where both interventions influence saturable inflammatory outputs or push autophagy in different directions in different cell types.

### 4.3 Testable predictions of the convergence hypothesis

The convergence hypothesis is falsifiable only if interaction and mechanism are analysed separately. At each functional domain, the combination may outperform both monotherapies, match the stronger monotherapy, fall between monotherapies, or worsen an outcome. These patterns should be prespecified rather than interpreted *post hoc* as synergy.

For fibrogenesis, interaction should be assessed with histological fibrosis, hydroxyproline or collagen content, alpha-SMA, collagen I, and, where feasible, cell-resolved HSC measurements. For redox biology, Nrf2 should be measured as an electroacupuncture-supported pathway, while ROS, lipid peroxidation, and NLRP3-associated injury provide broader shared outcomes; absence of saikosaponin-driven Nrf2 activation would refute an identical-node model without refuting functional redox overlap. For inflammation, hepatic macrophage phenotypes, NF-kappaB/NLRP3 activity, cytokines, and neural-dependence tests are needed to distinguish direct pharmacology from somatosensory-autonomic effects. For metabolism and autophagy, lipid flux, AMPK activity, autophagosome formation, lysosomal completion, and cell-type-specific flux should be measured because static marker levels can misclassify blocked versus enhanced autophagy.

Gut-liver measurements should be integrated into the same experiment rather than added as an isolated descriptive analysis. Microbiome composition, targeted bile-acid profiles, intestinal permeability, portal inflammatory markers, and hepatic endpoints can identify correlation. Mediation requires perturbation, such as microbiota depletion or transfer, barrier-focused intervention, or receptor-level testing. This design would determine whether a gut-liver route explains any interaction or merely accompanies changes produced elsewhere.

A suitable first study would use a clinically relevant metabolic-fibrosis model and a prespecified 2 x 2 factorial structure: vehicle/sham, a defined purified saikosaponin or formulation, a reproducible and fully reported acupuncture-related protocol, and the combination. A second injury model could test generalisability. Randomisation, blinded outcome assessment, dose-ranging, appropriate sham procedures, and formal interaction terms are essential. Pharmacokinetics and safety should be collected in the same animals. Only a result reproduced across dose or timing conditions, accompanied by mechanistic perturbation where possible, would support progression beyond proof of concept.

### 4.4 Key evidence gaps and future research needs

The research sequence should begin with selection of a lead congener and formulation, followed by repeat-dose toxicology, safety pharmacology, and drug-interaction assessment. Standardized acupuncture parameters should be reported according to STRICTA and fixed before confirmatory experiments (MacPherson et al., 2010). Factorial animal studies should then estimate interaction and test the four functional domains plus the gut-liver interface. Human development would require early-phase pharmacokinetic and safety evaluation before efficacy trials. Clinical endpoints must match the construct being studied: MRI-PDFF measures steatosis; elastography estimates liver stiffness rather than directly proving histological fibrosis reversal; biopsy-based histology may serve as an intermediate endpoint when justified; and decompensation, transplantation, hepatocellular carcinoma, and mortality are clinical outcomes requiring larger and longer studies. The present evidence does not justify bypassing these stages or presenting combined treatment as clinically ready (Figure 2).

**FIGURE 2:**
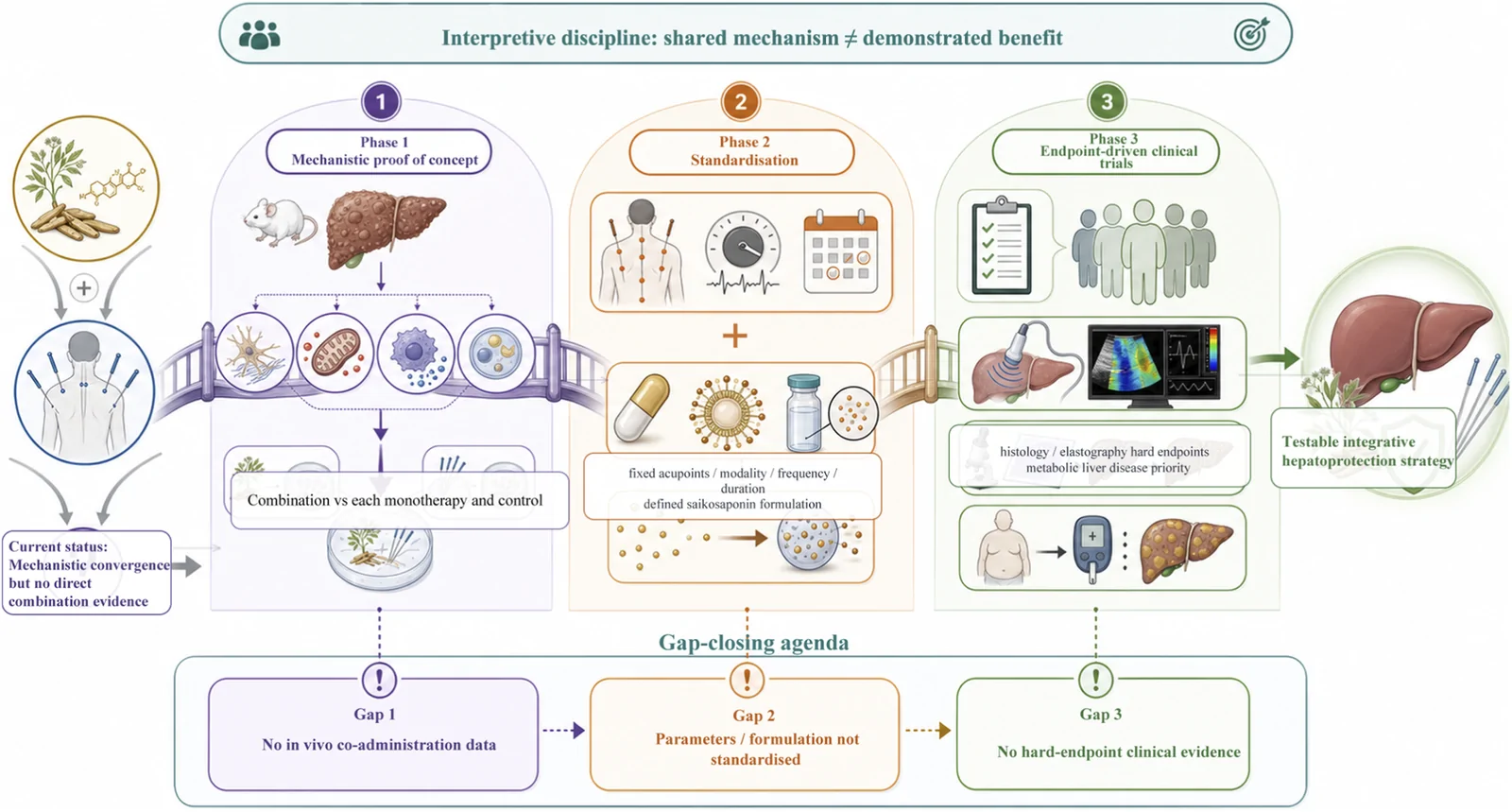
Staged research roadmap for testing combined saikosaponin and acupuncture treatment. Initial work should select a lead congener and formulation and define a reproducible, modality-specific acupuncture protocol. Factorial preclinical studies should estimate treatment interaction while measuring mechanism, pharmacokinetics, gut-liver variables, and safety. Repeat-dose toxicology, safety pharmacology, and drug-interaction assessment should precede early-phase human pharmacokinetic and safety studies. Efficacy trials should be considered only after an acceptable benefit-risk profile is established. Endpoints should match the construct: MRI-PDFF for steatosis, elastography for noninvasive stiffness assessment, histology when biopsy is justified, and clinical outcomes in adequately powered longer-term studies.

### 4.5 Multi-omics, network pharmacology and computational approaches

The programme outlined above is demanding, and computational and systems methods can make it more tractable. Network pharmacology treats a compound not as a single-target agent but as a modifier of an interaction network, an approach that suits a multi-component intervention and a multi-node mechanism (Hopkins, 2008). It has already been applied to Bupleurum-containing formulas. Integrated network-pharmacology and multi-omics analysis of Chaihu-Shugan-San identified overlapping targets in liver fibrosis (Xie et al., 2022), and a combined network and experimental study of Xiaoyao-San mapped its action against steatohepatitis-related fibrosis onto STAT3, NF-κB and PPAR signalling (Yan et al., 2024). These formula-level studies are methodological templates, not evidence for single saikosaponins, and the distinction between a whole prescription and an isolated congener must be kept in view.

Multi-omics and computational methods can prioritize experiments but cannot establish interaction by themselves. Transcriptomic, lipidomic, metabolomic, bile-acid, and microbiome profiles can locate candidate response patterns across the four domains and the gut-liver interface. Network or machine-learning analyses may nominate dose, timing, or biomarker combinations, but their outputs depend on heterogeneous datasets and require prospective experimental validation. They should be used to formulate candidate interaction patterns, not to label targets as synergistic before a factorial experiment.

## 5 Discussion and future perspectives

This review supports a limited but useful conclusion. Selected saikosaponins and defined acupuncture-related modalities show partially overlapping downstream effects in fibrogenic, redox, inflammatory, and metabolic-autophagic biology, with a possible gut-liver interface. The evidence is structurally asymmetric: saikosaponin data are predominantly molecular and preclinical and are constrained by congener specificity, model limitations, bioavailability, toxicity, and drug-interaction concerns; acupuncture data are modality-dependent, with stronger human evidence for metabolic outcomes than for fibrosis and stronger mechanistic evidence for electroacupuncture or moxibustion than for manual needling. Nrf2 is not an equivalently established shared pathway, and neither AMPK nor the gut-liver axis is proven as a common causal mechanism. No direct combination study was identified. The appropriate next step is therefore a staged program of standardized factorial experiments, mechanistic perturbation, pharmacokinetic and toxicological assessment, and only subsequently early-phase human safety research. Combined saikosaponin and acupuncture treatment remains a testable research hypothesis rather than an established hepatoprotective therapy.
